# A New Facile Synthesis of D_4_-Pterosin B and D_4_-Bromopterosin, Deuterated Analogues of Ptaquiloside 

**DOI:** 10.3390/molecules17055795

**Published:** 2012-05-16

**Authors:** Mohamed Attya, Monica Nardi, Antonio Tagarelli, Giovanni Sindona

**Affiliations:** Department of Chemistry, University of Calabria, 87036 Arcavacata di Rende (Cosenza), Italy

**Keywords:** natural products, total synthesis, isotopic labelling, ptaquiloside, pterosin B, d_4_-pterosin, d_4_-bromopterosin

## Abstract

Ptaquiloside (Pta) is a potent carcinogen present in bracken fern and in soil matrices, that can potentially leach to the aquatic environment. More recently its presence in the milk of different farm animals has been reported. Pterosin B (Ptb) and bromopterosin (BrPt) represent the most convenient analogues in the detection of ptaquiloside by mass spectrometry. Pterosin sesquiterpenes are also involved in many patented biomedical protocols. In this work we introduce a new and convenient approach to the synthesis in three steps and more than 80% yield of d_4_-pterosin B (d_4_-Ptb) and d_4_-bromopterosin (d_4_-BrPt), useful as internal standards in the quantification of ptaquiloside.

## 1. Introduction

**Figure 1:**
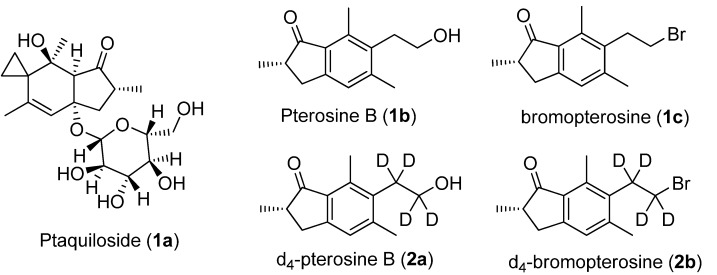
The structure of ptaquiloside, pterosin B, bromopterosin, d_4_-pterosin B, and d_4_-bromopterosin.

Ptaquiloside (Pta, **1a**, Figure 1) is, from a toxicological point of view the most intriguing secondary metabolite found in bracken and responsible for more than 50% of the carcinogenic activity of bracken fern [1]. Bracken fern is currently consumed by farm animals and causes a number of well-known syndromes in domestic animals [2]. In large ruminants, chronic enzootic haematuria, the clinical expression of multiple neoplasia of the urinary bladder, occurs. Pta has been shown in laboratory experiments to be carcinogenic [3], and it is co-responsible for urinary bladder tumours in cows. Pta may represent a pollutant of foods derived from cattle [4]. A significant increase in gastric cancers in humans who spend their childhood in bracken-infested areas has been demonstrated [5], and milk has been proposed as the carrier, in which Pta would not be destroyed by pasteurisation. Pterosin compounds are also involved in many patented biomedical protocols for treating diabetes and obesity [6,7]. Ptaquiloside is unstable in aqueous solution as it undergoes hydrolytic decomposition under acid as well as alkaline conditions. Under alkaline conditions, the reaction products are first ptaquilosin and then an unstable dienone formed after liberation of D-(+)-glucose [1,8]. Under acid conditions aromatization takes place and the reaction product is mainly pterosin B. Ptaquiloside is rather stable at neutral pH. Pterosin B (Ptb **1b**; Figure 1) is stable under both acid and alkaline conditions [9], while ptaquilosin and the dienone are stable only under alkaline conditions [1,8,10].

Quantification is most commonly carried out by HPLC by means of external standards. Under some circumstances, ptaquiloside is converted to pterosin B before analysis, since it is more stable than ptaquiloside and has stronger UV absorption. The indirect detection of ptaquiloside is preferred for two reasons: the conversion to pterosin B results in an approximate doubling of the UV response and will hence lower the limit of detection, overcoming problems associated with ptaquiloside handling. LC-UV methods however, lack of specificity and false positives may arise from substances with similar chromatographic and spectroscopic properties. Mass spectrometric detection, on the contrary, provides the required uniqueness in structure identification and enough sensitivity and accuracy in the analytical application. A mass spectrometric approach was recently successfully applied. It is based on the transformation of ptaquiloside to bromopterosin (BrPt, **1c**; Figure 1) or methoxypterosin followed by a GC-MS and stable isotope dilution assays using d_2_-bromopterosin as internal standard [11,12]. More recently, a sensitive LC-MS/MS method for quantifying Pta in soil and groundwater based on the transformation of ptaquiloside to pterosin B has been published [13]. This work reports a new method for the synthesis of the ptaquiloside’s deuterated analogues d_4_-pterosin B and d_4_-bromopterosin (**2a**, **2b**, Figure 1).

## 2. Results and Discussion

**Scheme 1:**
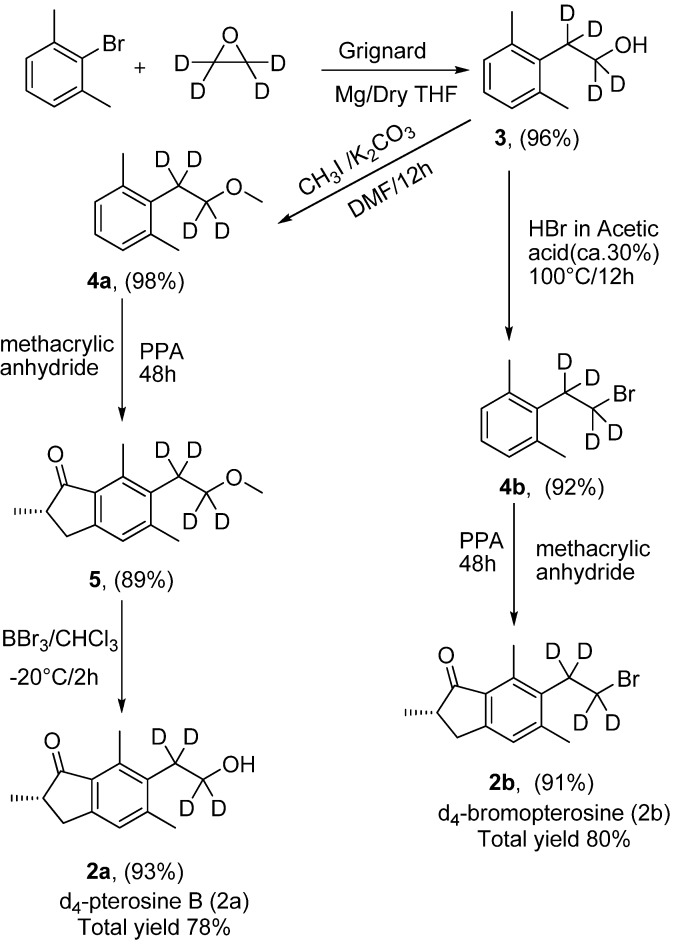
The synthesis of d_4_-pterosin B and d_4_-bromopterosin.

The aim of this work was to synthesise deuterated analogues to be used as internal standards in the quantification of Pta by the isotope dilution method. Several synthetic routes have been proposed since 1974 to prepare pterosin derivatives [14,15,16,17,18], but the drawbacks of all those methods are the low yield, not more than 50%, and the high number of steps, not less than six. To the best of our knowledge only one method has been proposed for the synthesis of deuterated analogues of the Pta [19], d_2_-pterosin B and d_2_-bromopterosin, but the overlap between the ^81^Br of bromopterosin and the ^79^Br of d_2_-bromopterosin isotopic peaks may prevent the use of the di-deuterium labelled compound as internal standard. In the previous approach, d_2_-bromopterosin was obtained in eight steps with less than 60% overall yield. In the procedure now proposed (Scheme 1) d_4_-bromopterosin (**2b**; Figure 1) is obtained in three steps with 80% overall yield, and d_4_-pterosin B (**2a**; Figure 1) in four steps with 78% overall yield. The presented synthetic protocol has many advantages over all the other known methods for the synthesis of pterosin B and deuterated pterosin B, as it presents the highest yield and the lowest work load. Our synthetic route is based on a successful modification of the previous proposed methods [18,19,20], in which the tetradeuteroalcohol **3** was obtained in only one step in a very good yield, and the final products could also be obtained in good yield by using the methacrilic anhydride and PPA as condensing agent in a one-step reaction [19], avoiding the low yield which was generally reported for the last step. The use of the available deuterium labelled compounds ethylene oxide-d_4_ as a reactant has provided high isotope purity of the final product [21], comparing with the labelling of the whole compound by inserting of deuterium atoms.

The key step in our procedure is a simple Grignard reaction between 2,6-dimethylbromobenzene and ethylene oxide-d_4_ [18]; by this simple reaction we could obtain the tetradeuterated alcohol (2-(2,6-dimethylphenyl)ethanol-1,1,2,2-d_4_ (**3**) in only one step and a yield of 96%. The tetradeuterated substrate **3** is the starting substrate for obtaining our desired products d_4_-bromopterosin and d_4_-pterosin. For the synthesis of d_4_-bromopterosin, the tetradeuterated alcohol was brominated directly using HBr in acetic acid [22], to give 2-(2-bromoethyl-1,1,2,2-d_4_)-1,3-dimethylbenzene (**4b**), which was subjected to a condensation reaction using methacrylic anhydride and polyphosphoric acid to give the final product d_4_-bromopterosin in a total yield of 91%. D_4_-ptetosin was also obtained with a total yield of 78% by the application of the same condensation reaction on d_4_-methoxypterosin **4a**, followed by a demethylation reaction (Scheme 1) [23]. The proposed strategy demonstrates a new total synthesis for d_4_-bromopterosin and d_4_-pterosin. The usefulness of this strategy can be attributed not only to the synthesis of ptaquiloside’s deuterated analogues, but also for the synthesis of pterosin B itself, in which it demonstrated the highest yield and the lowest work load. 

## 3. Experimental

### General

Column chromatography was performed on silica gel 60 (Merck, 70-230 mesh). Optical rotations were measured on a Jasco DIP-1000 12 polarimeter equipped with a sodium lamp (589 nm) and a 10-cm microcell. ^1^H-NMR (300 MHz) and ^13^C-NMR (75 MHz) spectra of samples dissolved in CDC1_3_ were recorded on a Bruker DPX Avance 300 spectrometer at 25 °C; TMS was used as internal standard. IR spectra were recorded on a Perkin-Elmer Paragon 1000 PC FT-IR spectrometer. GC-MS spectra were obtained with a Shimadzu QP-2010 GC-MS apparatus (ionization voltage 70 eV). All reactions were analyzed by TLC on silica gel 60 F_254_ and by GLC on a Shimadzu GC-2010 gas chromatograph and capillary columns purity). 2,6-Dimethylbromobenzene, ethylene oxide-d_4_, iodomethane, HBr in acetic acid (*ca.* 30% ), methacrylic anhydride, BBr_3_, and polyphosphoric acid (PPA) are commercially available and were used as received (Sigma Aldrich).

*2-(2,6-Dimethylpheny1)ethanol-1,1,2,2-d_4_* (**3**). To a suspension of magnesium turnings (0.73 g, 30 mmol) in THF (5 mL), maintained under N_2_ and under reflux, was added slowly a solution of 2,6-dimethylbromobenzene (5 g, 27 mmol) in dry THF (30 mL) during 15 min. When the spontaneous reaction had ceased the mixture was refluxed for an additional 30 min. The water condenser was replaced by a liquid nitrogen and alcohol condenser (−20 °C). A well-cooled solution of ethylene oxide-d_4_ (4 mL, 3.58 g, 75 mmol) in dry THF (10 mL) was added during 5 min, then the mixture was refluxed for 2 h, keeping the condenser temperature always below −20 °C. After cooling, the dark coloured oil was added to ice water (75 mL) and concentrated HCl (5 mL). The two layers were separated and the aqueous phase was extracted with ether (3 × 30 mL). The combined organic phases were washed with brine, and then dried over Na_2_SO_4_. After filtration of the mixture and removal of the solvent by rotary evaporation, the residue was purified by column chromatography (silica gel, hexane–EtOAc, 9:1); this gave 2-(2,6-dimethylpheny1)ethanol-d_4_ (**3**). Yield: 4.01 g (26.00 mmol, 96%); colourless viscous oil; IR (film): ν = 3330 (s), 1468 (s), 1298 (w), 1039 (s) cm^−1^; ^1^H-NMR (CDCl_3_): *δ* = 6.92–7.01 (m, 3 H, Ph), 2.35 [s, 6 H, 2(C*H_3_*)]; ^13^C-NMR (CDCl_3_): *δ* = 136.9, 128.2, 126.2, 120.1, 60.6, 20.0, 15.8; GC-MS (EI, 70 eV): *m/z* = 154 [M^+^] (20), 121 (100), 107 (15), 92 (20).

*2-(2-Methoxyethyl-1,1,2,2-d_4_)-1,3-dimethylbenzene* (**4a**). A mixture of the alcohol **3** (1.54g, 10 mmol), iodomethane (3.1 mL, 7.08 g, 50 mmol), anhydrous K_2_CO_3_ (3.45 g, 25 mmol), and DMF (20 mL) was stirred at room temperature for 12 h. After the reaction was completed the reaction mixture was diluted with water (50 mL) and extracted with chloroform (3 ° 50 mL). The combined organic phases were washed with water, brine (100 mL each), and then dried over Na_2_SO_4_. After filtration of the mixture and removal of the solvent by rotary evaporation, the residue was purified by column chromatography (silica gel, hexane–EtOAc, 95:5); to give methyl 2-(2,6-dimethylpheny1)ethyl ether-d_4_ (**4a**). Yield: 1.66 g (9.88 mmol, 98.8%); colourless oil; IR (film): ν = 2880 (s), 1100 (s) cm^−1^; ^1^H-NMR (CDCl_3_): *δ* = 6.92–7.01 (m, 3 H, Ph), 3.25 (s, 3 H, OC*H_3_*), 2.35 [s, 6 H, 2(C*H_3_*)]; ^13^C-NMR (CDCl_3_): *δ* = 136.9, 128.2, 126.2, 120.1, 60.6, 55.4, 20.0, 15.8; GC-MS (EI, 70 eV): *m/z* = 168 [M^+^] (25), 154 (20), 121 (100), 107 (15), 92 (20).

*2-(2-Bromoethyl-1,1,2,2-d_4_)-1,3-dimethylbenzene* (**4b**). To a solution of the alcohol **3** (1.54 g, 10 mmol) in glacial acetic acid (4 mL) was added HBr (6 mL, 30% HBr in acetic acid), and the mixture was heated at 100 °C in a sealed tube for 12 h. After cooling to room temperature, the reaction mixture was poured onto a cold solution of saturated NaHCO_3_ (50.00 mL), and the mixture was extracted with chloroform (3 × 30 mL). The combined organic phases were washed with brine, and then dried over Na_2_SO_4_. After filtration of the mixture and removal of the solvent by rotary evaporation, the residue was purified by column chromatography (silica gel, hexane–EtOAc, 95:5); to give 1-bromo-2-(2,6-dimethylpheny1)ethyl-d_4_ (**4b**). Yield: 1.99 g (9.21 mmol, 92%); colourless oil; IR (film): ν = 2880 (s), 1440 (s), 1298 (s) cm^–1^; ^1^H-NMR (CDCl_3_): *δ* = 6.92–7.01 (m, 3 H, Ph), 2.35 [s, 6 H, 2(C*H_3_*)]; ^13^C-NMR (CDCl_3_): *δ* = 136.9, 128.2, 126.2, 120.1, 29.6, 20.0, 15.8; GC-MS (EI, 70 eV): *m/z* = 218 [M^+^+2] (14), 216 [M^+^] (15), 137 (80), 121 (100), 93 (30).

*6-(2-Methoxyethyl-1,1,2,2-d_4_)-2R,5,7-trimethyl-2,3-dihydro-1H-inden-1-one* (d_4_-methoxypterosin, **5**). Methacrylic anhydride (1.16 mL, 1.19 g, 7.70 mmol) was added to a suspension of methyl 2-(2,6-dimethylpheny1)ethyl ether-d_4_ (**4a**, 0.875 g, 5.20 mmol) in PPA (30 g). Because of the high viscosity of the PPA the reaction mixture was stirred by mechanical stirrer for 48 h at room temperature, and after diluted with ice water (50 mL), and extracted with chloroform (3 × 30 mL). The combined organic phases were washed with brine, and then dried over Na_2_SO_4_. After filtration of the mixture and removal of the solvent by rotary evaporation, the residue was purified by column chromatography (silica gel, hexane–EtOAc, 96:4); to give d_4_-pterosin methyl ether **5**. Yield: 1.1 g (4.66 mmol, 89.6%); colourless oil; [α]^25^_D_ −31 (*c* 5 mg/mL); IR (film): ν = 1740 (m), 1700 (s), 1675 (w), 1590 (s) cm^−1^; ^1^H-NMR (CDCl_3_): *δ* = 7.0 (s, 1 H, ph), 3.51 (dd, *J* = 8.0, 16.4 Hz, 1 H, C*H*), 3.28 (s, 3 H, O*CH_3_*), 2.59–2.77 (m, 2 H, C*H_2_*), 2.35 (s, 3 H, 5-C*H_3_*), 2.24 (s, 3 H, 7-C*H_3_*), 1.26 (d, *J* = 8.0 Hz, 3 H, 2-*CH*_3_); ^13^C-NMR (CDCl_3_): *δ* = 210.1, 152.2, 141.2, 137.3, 134.1, 131.3, 122.8, 60.9, 55.1, 42.0, 33.5, 31.7, 20.1, 16.3, 13.5; GC-MS (EI, 70 eV): *m/z* = 236 [M^+^] (30), 221 (50), 189 (100).

*6-(2-Hydroxyethyl-1,1,2,2-d_4_)-2R,5,7-trimethyl-2,3-dihydro-1H-inden-1-one* (d_4_-pterosin B, **2a**). To a solution of d_4_-pterosin methyl ether **5** (1.08 g 4.60 mmol) in chloroform (10 mL), was added BBr_3_ (1.75 g, 0.67 mL, 7 mmol) at −20 °C, the solution was stirred at the same temperature for 2 h. The reaction mixture was added to a saturated solution of NaHCO_3_ (100 mL), and extracted with chloroform (3 × 50 mL). The combined organic phases were washed with brine, and then dried over Na_2_SO_4_. After filtration of the mixture and removal of the solvent by rotary evaporation, the residue was purified by column chromatography (silica gel, hexane–EtOAc, 95:5); to give d_4_-pterosin (**2a**). Yield: 0.96 g (4.32, 94%); mp. 90–92 °C; IR (KBr): ν = 3410 (s), 1736 (s), 1668 (s), 1590 (s), 1040 (s) cm^−^^1^; ^1^H-NMR (CDCl_3_): *δ* = 7.0 (s, 1 H, ph), 3.65 (br s, exchangeable, l H, O*H*), 3.53 (dd, *J* = 8.0, 16.4 Hz, 1 H, C*H*), 2.56–2.76 (m, 2 H, C*H_2_*), 2.35 (s, 3 H, 5-C*H_3_*), 2.23 (s, 3 H, 7-C*H_3_*), 1.27 (d, *J* = 8.0 Hz, 3 H, 3-*CH*_3_); ^13^C-NMR (CDCl_3_): *δ* = 210.1, 152.2, 141.2, 137.3, 134.1, 131.3, 122.8, 60.9, 42.0, 33.5, 31.7, 20.1, 16.3, 13.5; GC-MS (EI, 70 eV): *m/z* = 222 [M^+^] (40), 207 (70), 189 (100).

*6-(2-Bromoethyl-1,1,2,2-d_4_)-2R,5,7-trimethyl-2,3-dihydro-1H-inden-1-one* (d_4_-bromopterosin, **2b**). Methacrylic anhydride (1.03 mL, 1.05 g, 6.80 mmol) was added to a suspension of methyl 2-(2,6-dimethylpheny1)ethyl ether-d_4_ (**4a**, 1.00 g, 4.60 mmol) in PPA (30 g). The reaction stirred by mechanical stirrer for 48 h at room temperature, and after diluted with ice water (50 mL), and extracted with chloroform (3 × 30 mL). The combined organic phases were washed with brine, and then dried over Na_2_SO_4_. After filtration of the mixture and removal of the solvent by rotary evaporation, the residue was purified by column chromatography (silica gel, hexane–EtOAc, 95:5); to give d_4_-bromopterosin **2b**. Yield: 1.19 g (4.19 mmol, 91%); yellow crystals; mp. (108–109 °C); [α]^25^_D_ −21 (*c* 5 mg/mL); IR (KBr): ν = 2880 (w), 1736 (s), 1668 (s), 1590 (s), 1440 (w), 1040 (s) cm^−1^; ^1^H-NMR (CDCl_3_): *δ* = 7.0 (s, 1 H, ph), 3.51 (dd, *J* = 8.0, 16.4 Hz, 1 H, C*H*), 2.60-2.76 (m, 2 H, C*H_2_*), 2.37 (s, 3 H, 5-C*H_3_*), 2.26 (s, 3 H, 7-C*H_3_*), 1.29 (d, *J* = 8.0 Hz, 3 H, 2-*CH*_3_); ^13^C-NMR (CDCl_3_): *δ* = 210.1, 152.2, 141.2, 137.3, 134.1, 131.3, 122.8, 42.0, 33.9, 33.6, 20.1, 15.3, 15.1, 13.6; GC-MS (EI, 70 eV): *m/z* = 286 [M^+^+2] (50), 284 [M^+^] (50), 205 (100), 189 (50).

## 4. Conclusions

In conclusion, we have developed a new facile and convenient total synthesis of d_4_-pterosin B in four steps with a total yield of 78%, and d_4_-bromopterosin in three steps with a total yield of 80%. The synthesized compounds can be used as internal standards for the assay of pterosin sesquiterpenes and analogues in food and biological fluids and could provide the basis for the formation of labelled analogues of other members of this peculiar family of natural compounds.
